# Characterizing Middle School Students' Physical Literacy Development: A Self-Determination Theory-Based Pilot Intervention in Physical Education

**DOI:** 10.3389/fspor.2021.809447

**Published:** 2022-01-12

**Authors:** Yang Liu, Senlin Chen

**Affiliations:** ^1^Department of Physical Education, Wuhan University of Technology, Wuhan, China; ^2^School of Kinesiology, Louisiana State University, Baton Rouge, LA, United States

**Keywords:** canadian assessment of physical literacy, curriculum and instruction, learning, secondary physical education, self-determination theory

## Abstract

**Purpose:** Positive youth development (PYD) can be achieved through effective and purposeful instructions in physical education (PE) and other relevant experiences both in and beyond schools. Students' PYD is associated with their physical literacy (PL) development, which has become a primary emphasis of PE, especially in the United States, in recent years. This study aimed to (a) characterize middle school students' physical literacy (PL) and (b) capture their PL developing trajectories in light of receiving a self-determination theory (SDT)-based pedagogical workshop, with the long-term vision on PYD.

**Methods:** Participants (*N* = 226) completed the Canadian Assessment of Physical Literacy (CAPL-2) in physical education (PE). A subsample (*n* = 49) received four workshop sessions over 8 weeks; and completed the CAPL-2 and participated in focus group interviews before and after the workshop.

**Results:** Both boys and girls' CAPL-2 scores were in the “progressing” stage. Significant differences in PL and PL domains were observed by gender, grade, socioeconomic status (SES), body mass index (BMI), and race/ethnicity. The low PL group showed improvements in PL and PL domains. Interview data delineated positive PL developing trajectories for physical activity (PA) type, frequency, and intensity; perceived motives; and participation barriers.

**Conclusion:** PL is a dynamic state that can be improved through purposeful PE. Future work should examine the effect (and implementation) of opportunities in (e.g., PE) and beyond schools (e.g., youth sports programs) to ultimately advance PYD.

## Introduction

Positive youth development (PYD) is a strength-based approach to fostering youth to become contributing members of the societies, through emphasizing competence, confidence, character, connection, and caring (Lerner, 2009). PYD has traditionally been a major topic of interest among practitioners, researchers, and stakeholders outside of the school setting (e.g., youth sports, non-profit organizations). School-based programs such as physical education (PE) could contribute to PYD by equipping youth with essential life skills and psychosocial and behavioral attributes, although research evidence from the PE setting is limited (Wright and Li, 2009; Weiss, 2011; Santos et al., 2019). The goal of PE, in the United States (U.S.) in particular, is to nurture “physically literate individuals” (Society of Health Physical Educators (SHAPE) America, 2013; The Aspen Institute, 2015). Physical literacy (PL) is a concept that has received global attention in research, practice, and policy discourses in recent years (Roetert and Jefferies, 2014; The Aspen Institute, 2015). In fact, PL development is associated with PYD through relevant out-of-school opportunities and school PE experiences (Allan et al., 2017). This present study examined middle school students' PL development from the perspective of PE with the long-term vision on PYD. Effective PE pedagogy has the potential to foster students' PL development through lived experiences both within and beyond the PE class (Liu and Chen, 2020a). Whitehead (2013, p. 29) defines PL as “the motivation, confidence, physical competence, knowledge and understanding to value and take responsibility for engagement in physical activities for life.” Developing PL involves the physical, affective and cognitive domains (Whitehead, 2010), with others also including additional domains such as behavioral [CAPL-2; Healthy Active Living and Obesity Research Group (HALO), 2017] and social domains [Australian Institute of Sport (AIS), 2019]. Other definitions of PL also exist (Liu and Chen, 2020a), but experts have reached a consensus that the ultimate goal of PL development is lifelong physical activity (PA) engagement (Whitehead, 2010, 2013). However, few empirical studies in the U.S. have investigated middle school students' comprehensive PL and fewer have attempted to foster PL using theory-based interventions.

A few studies have investigated students' PL by age, gender, body mass index (BMI), and socioeconomic status (SES) using the Canadian Physical Literacy Assessment (CAPL-2; Longmuir et al., 2015; Bélanger et al., 2018; Delisle Nyström et al., 2018; Dutil et al., 2018; Law et al., 2018; Tremblay et al., 2018b). These studies, mostly took place outside of the U.S., reported a low level of PL ranging from 59.3 to 64.9% for children of 8–12 years old. The CAPL-2 [Healthy Active Living and Obesity Research Group (HALO), 2017] is the only tool to date capable of determining a PL composite score. PL scores appeared to favor boys (Cohen's *d* = 0.07–0.20; Bélanger et al., 2018; Dutil et al., 2018; Tremblay et al., 2018b), older students (e.g., *d* = 0.27; Dutil et al., 2018; Tremblay et al., 2018b), and students of healthy BMI (*d* = 0.30; Delisle Nyström et al., 2018). Exploring the differences in PL and PL domains across demographic groups is significant for designing and tailoring PL interventions. To date, rarely have studies explored the demographic differences in PL in the US.

Developing PL in youth holds significance to public health as well as education (Castelli et al., 2014; Whitehead et al., 2018). It is critical to equip students with competence and confidence needed for lifetime PA participation. Lacking of PA is associated with morbidities, which requires counter strategies in schools and other settings to nourish PL for active living (Dumith et al., 2011). Developing PL is educationally meaningful (Whitehead et al., 2018). PE is a primary, though not the sole, setting to foster PL (Castelli et al., 2014; The Aspen Institute, 2015). Interventions do exist to promote PL in PE context but mainly by working with teachers through professional development (Durden-Myers, 2020). Few interventional studies have attempted to promote students' PL development (McGrane et al., 2018), although several studies targeted improving discrete PL components. The effective interventions often followed a robust theoretical framework such as the Social Ecological Model (Castelli et al., 2014; Bélanger et al., 2016), or Health Belied Model (Castelli et al., 2014). These studies mostly used quantitative rather than qualitative or mixed methods (Kiez, 2015; Mateus et al., 2015; Bélanger et al., 2016; George et al., 2016; Johnstone et al., 2017; Lavery et al., 2017; McGrane et al., 2018; Wainwright et al., 2018). However, as theorized by Whitehead (2001, 2013), PL is a state of embodied capability. Therefore, a mixed methods approach with a longitudinal perspective may be better suited to capture PL developmental trajectory. These existing interventions have further suggested a minimum of 4–6 weeks to yield significant effects (George et al., 2016).

Developing PL requires adaptive motivation, an appropriate internal drive capable of activating individuals' valuing to move (Linnenbrink-Garcia et al., 2016). Whitehead (2010, p. 12) proposed that PL “can be described as a disposition characterized by the motivation to capitalize on innate movement potential to make a significant contribution to the quality of life.” Chen (2015) contended that self-determined motivation is foundational to the appropriate functioning of PL components such as knowledge, skillfulness, and confidence. PE provides students with the opportunities to learn attributes of motivation for PL-related outcomes (Chen, 2015). McClelland (2013) proposed to foster motivation for PL development in PE as guided by self-determination theory (SDT). SDT features a behavioral regulation continuum that characterizes motivation from amotivation, external regulation, and introjected regulation to identified regulation, integrated regulation, and intrinsic motivation (Deci and Ryan, 1985). A person's motivation level is a reflection of the degree to which a behavior is internalized, and determined by fulfillment of the needs for competence, relatedness, and autonomy. When these needs are satisfied in a supportive environment, motivation is expected to grow toward the more self-determined forms (i.e., identified and integrated regulations, intrinsic motivation). McClelland (2013) posited that factors such as motivations for fun experiences, knowledge learning, movement skills development, movement competence, and relatedness would significantly influence PL development. The role of PE within the school context is essentially through providing learning and motivational opportunities (Kouneiher et al., 2009). SDT-based motivation is at the heart of PL development across the physical, cognitive, affective, and behavioral domains in PE, which is believed to reinforce students' multifaceted learning activities in PE (McClelland, 2013; Chen, 2015). Therefore, in this study, an SDT-based pedagogical workshop was designed as intervention to foster students' fun experience, knowledge learning, fundamental skills, and valuing for becoming more physically literate. The SDT-oriented, needs-supportive learning environment emphasized fulfilling students' perceptions of competence, relatedness, and autonomy.

### Research Purposes

As synthesized above, little empirical evidence is available to inform middle school students' PL development in the U.S. and few interventions are documented in the literature. In this study, we aimed: (a) to characterize middle school students' PL development as well as its distribution patterns by gender, grade, SES, weight status, race, and ethnicity; and (b) to capture PL journeys in light of receiving a SDT-guided pedagogical workshop.

## Methods

### Research Setting and Participants

To address the research purposes, the sequential intervention mixed methods design was used, as we were interested in first ascertaining adolescents' PL level, and then based on the observed PL developmental stage imposing the workshop intervention to render PL gains for both high and low PL-performed adolescents. The study took place in one suburban public middle school located in a Southeastern U.S. state. The school enrolled 483 students in sixth, seventh, and eighth grades at the time of this study. Students to teacher ratio was 18.58, and White (*n* = 254) was the primary race followed by Black (*n* = 197) and others (*n* = 32). The school had roughly even number of boys (*n* = 224) and girls (*n* = 259). Most of the students (61.7%) were eligible for free or reduced-price lunch. The PE followed the multi-activity model and taught by two certified PE teachers in the gymnasium with six classes scheduled in the morning and six in the afternoon. Students in sixth and seventh grades attended PE together on every another day.

A convenience sample of 226 students (boys = 46.9%; age = 12.16 ± 0.73; from eight classes) in sixth (*n* = 132; boys = 57.6%, age = 11.72±0.49) and seventh (*n* = 94; boys = 31.90%, age = 12.70 ± 0.62) grades voluntarily participated in the study upon turning in parental consent and minors assent. Table 1 shows the characteristics of the sample. We used criterion-based purposeful sampling (“Beginning” vs. “Excelling” stages as assessed by CAPL-2) to select the sub sample to receive the workshop intervention (*n* = 49), where students were placed into low- (*n* = 26; CAPL-2 score = 52.06 ± 10.20) or high-performing (*n* = 23; CAPL-2 score = 78.27 ± 5.84) groups following prior criteria [Healthy Active Living and Obesity Research Group (HALO), 2017]. The participants were free of physical restrictions. The study protocol was approved by second author's Institutional Review Board and the school, while signed written parental consent and child assent were obtained.

**Table 1:** Characteristics of the sample.

**Variable name**	**Category**	**Frequency**	**%**
Gender			
	Male	106	46.7
	Female	121	53.3
Grade			
	Sixth graders	133	58.6
	Seventh graders	94	41.4
Ethnicity			
	Hispanic/Latino	12	5.3
	Not Hispanic/Latino	187	82.4
Race			
	American Indian or Alaska Native	6	2.6
	Asian	1	0.4
	African American/Black	70	30.8
	Native Hawaiian or other Pacific Islander	0	0
	White	90	39.6
	Two or more races	23	10.1
SES			
	Free lunch	106	46.7
	Reduced-price lunch	13	5.7
	Self-paid lunch	108	47.6
Weight status			
	Underweight	6	2.6
	Normal BMI	126	55.5
	Overweight or Obese	40	17.6
	Obese	47	20.7

*SES, socioeconomic status; BMI, body mass index*.

### The Pedagogical Workshop

Learning in high-low performing dyads creates heterogeneity (group differences) which, through appropriate scaffolding of the teacher (e.g., providing necessary supports), would enable the less capable learner to approach and close the *zone of proximal development* (potential room to improve) with the more capable learner (Vygotsky, 1978). Heterogeneous grouping is also encouraged to foster a more conducive motivational learning climate (Epstein, 1989). Each workshop session included two modules with the content and delivery based on the Heart PL model (McClelland, 2013), a framework that targets core motivation aspects most connected to helping nurturing PL achievement (McClelland, 2013). The motivational module was designed to foster PL development in behavioral (e.g., PA participation), physical (health-related fitness) and affective domains (confidence and motivation) through instruction, communication, and encouragement. The informational module addressed the cognitive (e.g., knowledge and understanding) and physical (e.g., motor skills) domains through instruction and demonstration. According to Whitehead (2010), being motivated to adopt a physically active lifestyle is essential to PL development. Empirically, the Heart PL Model depicted that motivation for fun (affective) effectively drive a student to achieve the ultimate goal of PL (being physically active), together with motivation from health-related fitness knowledge (cognitive), competence in fitness and fundamental movement skills (physical), and relatedness (McClelland, 2013). The SDT (Deci and Ryan, 1985) and the Heart PL model (McClelland, 2013) provided key theoretical guidance for us to design the pedagogical workshop modules. Table 2 shows the overview of the workshop sessions.

**Table 2:** Overview of the SDT-based workshop.

**Domains**	**Activities**	**Procedures**	**Targets**	**SDT-based supports**	**Goals**
Cognitive	Instructions and communications	Distribute handouts (four sessions); short review; present concepts related to PA, fitness and health.	Promote knowledge and understanding.	Support the need for perceived competence.	Competent in knowledge attainment, innovation, and transferability.
Physical	Explanations and demonstrations	Distribute handouts on motor skill tips; explain movement skills in real sport/exercise; provide strategies to overcome difficulties in skill performance.	Improve movement skills and capabilities to address skill-related challenges.	Support the need for perceived competence.	Achieve motor competence.
Behavioral	Communication and encouragement	Survey perceived fun and barriers in physical activities in the past week; have students recall active experiences; reinforce fun; help students address difficulties.	Reinforce fun experiences; foster predilection to seek fun; overcome barriers for activity participation; encourage intrinsic/integrated regulation.	Support the need for perceived competence.	Foster intrinsic motivation for PA.
Affective	Communication and encouragement	Survey perceived challenges and barriers in fitness-enhancing exercises and social activities; help them address difficulties with suggestions and encouragement.	Gain confidence and skills for socialization; understand the importance of fitness; stay motivated for PA.	Support the needs for relatedness and competence.	Become competent in socialization and motivated for health-related fitness.

The four workshop sessions (2 weeks pretest, 8 weeks intervention, and 2 weeks posttest) were implemented by a research assistant during regular PE classes between September and November in 2019. Each session lasted for 20–30 min across 16 lessons, starting with the motivational module followed by the informational module. The workshop occurred in the adjacent work room to the gym. After participants were seated, printed handouts were distributed. Activities to implement the motivational module included instruction, communication, and encouragement, where pedagogical skills (i.e., group discussion, interaction, and encouragement) were used to facilitate student engagement. To complete the series of activities during the motivational module, the student participants (a) shared with others their fun experiences, challenges/barriers, and social experiences related to physical activities; (b) received encouragement to participate in physical activities where they can seek fun; (c) worked together to provide possible solutions for others to overcome difficulties in performing these activities; and (d) received strategies for better socializing with others. These above activities were to reinforce fun experience, relatedness and competence in participating in PA, and valuing of exercises, which were in line with the affective, behavioral, and cognitive domains of PL.

The informational module was subsequently delivered with instruction and demonstration concerning the knowledge of health-related fitness and PA, tips to improve movement skills, health-related fitness, and behavioral strategies, which further addressed the cognitive, physical, and behavioral domains of PL. The taught knowledge was adopted from PE Metrics, CAPL-2 knowledge test, and youth PA guideline. For examples, one knowledge point was *Be Active: How long should we be physically active each day?* Students learned the recommended duration and intensity of PA. The tips to improve movement skills were demonstrated and explained for 13 strategies to improve separate performance in Canadian Agility and Movement Skill Assessment (CAMSA). For example, to improve the throwing skill, students were instructed (with demonstrations) on how to throw correctly with efficiency (e.g., with an opposition). For knowledge understanding, concepts of health-related fitness were explained to the participants, followed by strategies (i.e., workouts) to improve each fitness component. Content to promote an active lifestyle covered how to make age-appropriate decisions to foster skill learning and regular exercise participation. Strategies to maximize physical activities were also provided. The content for health-related fitness and knowledge informed participants of the importance of fitness-promoting exercises, and therefore enhanced their valuing of physical activities. Overall, informational module addressed the competency of physical and cognitive domains. Students turned in their completed worksheets in the end. Activities in informational module were to enhance competence in understanding, analyzing, and applying learned knowledge to a much broader and complicated physical setting, which address cognitive, physical, and behavioral domains.

### Instrumentation

#### PL Level

We used the CAPL-2 [Healthy Active Living and Obesity Research Group (HALO), 2017] to assess students' PL levels. CAPL-2 has separate assessments for the four domains (see Table 3; Francis et al., 2016; Gunnell et al., 2018). Scores across the four domains were aggregated to compute a composite score (100 in total). Based on the composite score, a PL level was assigned to interpret achievement: *beginning* (girls: <52.1; boys: <51.6), *progressing* (girls: 52.1–68.1; boys: 51.6–71.1), *achieving* (girls: 68.2–75.3; boys: 71.2–79.1), and *excelling* (girls: >75.3; boys: >79.1). CAPL-2 has shown sound reliability and validity in previous studies (Longmuir et al., 2015; Francis et al., 2016; Gunnell et al., 2018).

**Table 3:** CAPL-2 overview: domain, tools, and scoring.

**Domains (100 points)**	**Subdomains**	**Tools**	**Units**	**Points**
Physical (30 points)	Health-related fitness	– PACER (15/20 m)	Laps (count)	10
		– Isometric Plank Hold	Time (in second)	10
	Motor competence	– CAMSA (i.e., fundamental movement skill and agility)	14 levels	10
Behavioral (30 points)	Daily behavior	– Pedometer (daily step count)	Steps per day	25
		– Self-reported number of days per week participating in MVPA	Days	5
Cognitive (10 points)	Knowledge and understanding	– Physical literacy knowledge questionnaire	Number of items answered correctly	10
Affective (30 points)	Motivation and confidence	– Self-reported motivation and confidence questionnaire (predilection and adequacy, perceived competence, and internal motivation)	Likert scale	30

*CAMSA, Canadian agility and movement skill assessment; MVPA, moderate-to-vigorous physical activity; PACER, progressive aerobic cardiovascular endurance run*.

#### Weight Status and Sociodemographic Variables

Students' height (in meters) and weight (in kilograms) were measured by the PE teachers and research assistants (Seca 763 Physician Scales; Hogentogler & Co. Inc, Columbia, MD). When measured, students: (a) took off shoes and wore light sportswear; (b) stood on the scale with feet together; (c) faced back and looked forward; (d) kept knees and back straight; and (e) leaned their shoulders, buttocks, and back of the head against the stadiometer. Height and weight were recorded to the nearest hundredth. BMI percentile scores were subsequently calculated based on age, gender, height, and weight, which resulted in four weight status groups: underweight (<fifth percentile), normal BMI (fifth−85th percentile), overweight or obese (≥85th percentile), and obese [≥95th percentile; Centers for Disease Control and Prevention (CDC), 2018]. In addition, a questionnaire was used to collect sociodemographic data on gender, race, ethnicity, grade (i.e., sixth and seventh), and SES (i.e., free and reduced-price meal eligibility).

#### PL Journey

The subsample participated in the CAPL-2 assessments and semi-structured focus group interviews before and after the workshop to capture the potential changes of physical embodiment along students' trajectory to becoming more physically literate. These focus group interviews followed an interview guide with main and probe questions eliciting responses related to the participants' physical embodiment of PL development (Whitehead, 2013). Specifically, these questions asked about their participation, experiences, perceptions, and motivation of physical activities; as well as the changes over time. The conversations were recorded using an audio recorder (SONY, ICD–AX412, Sony Electronics Inc., San Diego, CA, USA). In addition, the first author took observation notes (55 school visits) and collected the completed worksheets, for data triangulation purpose. Students' PL journey as reflected in their lived physical experiences, was better characterized using a phenomenological approach to make meaning for the sub-group upon receiving the pedagogical workshop (Creswell, 2007, p. 57).

### Data Collection Procedures

Before data collection, a research assistant piloted the instruments and data collection procedures as well as all the workshop instructions. To ensure the workshop sessions be delivered as intended, we developed the lesson plans and ancillary written worksheets for all workshop sessions, and piloted them with adolescents. The second author trained the PE teachers to assist in data collection and workshop implementation. The baseline data collection for the quantitative part of the study started in August 2019. We started with collecting self-report data including a sociodemographic survey, knowledge test (cognitive domain), a PA behavior questionnaire (behavioral domain), and a motivation and confidence questionnaire [affective domain; Healthy Active Living and Obesity Research Group (HALO), 2017]. The surveys were distributed at the beginning of each PE lesson, and students were told that the survey had no right or wrong answers [Healthy Active Living and Obesity Research Group (HALO), 2017] and their responses would not affect school standings. Students independently completed the surveys, which took 14–18 min within one lesson. Subsequently, we distributed pedometers with a log sheet to each student and instructed them to properly wear the pedometer and record daily steps. The pedometers and completed log sheets were collected 8 days later, where the first day was deemed as a trial day. Within the following eight days, PACER, Isometric Plank Hold, and CAMSA were sequentially administered. Each assessment session accommodated 15 to 20 students. The trained teachers led the CAMSA assessment, which demonstrated moderate (*Kappa* = 0.41; McHugh, 2012) to high (*r* = 0.91) reliability. Posttest data collection (mid to late November) followed the same protocol as the pretest, but only on the subsample. Two semi-structured focus group interviews were conducted by the first author to gauge participants' PL journey, in light of receiving the pedagogical workshop. An interview guide was designed to facilitate conversations on PA behavior (e.g., types, frequency, intensity, time), barriers to PA participation, as well as physical embodiments. The focus group interviews involved two to eight students, which lasted for 22–32 min.

### Data Analysis

To address the first research purpose, descriptive statistics were conducted for PL overall and across the grouping factors, followed by inferential statistical analyses. MANOVA and ANOVA were performed to examine group differences by age and gender. Then, group differences by race/ethnicity, SES and weight status were analyzed using MANCOVA and ANCOVA, respectively, with gender and age as covariates (Longmuir et al., 2015; Lavery et al., 2017; Bélanger et al., 2018; Tremblay et al., 2018b). To address the second research purpose, repeated-measures ANCOVA was conducted to examine the time (pre vs. posttests) by group (high- vs. low-performing PL levels) interaction effect for PL domains. Partial-eta squared ($\eta_{p}^{2}$), Cohen's *d* (for *n* > 50), and Hedges' *g* (for *n* < 50) were reported as effect sizes. Assumption of homogeneity was examined using Box's *M* test and Levene's test. Welch's ANOVA was performed for data that showed heterogamous variance. For data with violation of normality assumption, Kruskal Wallis was performed. Alpha was set as 0.05.

To further address the second research purpose, we transcribed the interview data to verbatim and analyzed them on Nvivo11+ using content analysis. Both inductive and deductive approaches for data analysis were used to reveal participants' perception and motives/valuing of physical embodiments. For inductive analysis, the thematic analysis was conducted (Smith and Sparkes, 2016). Further, we conducted content analysis, which used the “latent approach” to create raw categories inductively and then embedded the categories in pre-existent constructs in a phenomenological way. Specifically, the pre-to-post PL change narratives of the participants were depicted in terms of PA patterns, motives (e.g., enjoyment), and barriers to physical activities. The first author immersed himself in the setting for a total of 55 school visits; and we triangulated the data collected from interviews, observations, and written records.

## Results

Table 4 shows the descriptive results of PL and PL domains for pretest by gender, grade, SES, BMI, race, and ethnicity. The CAPL-2 composite score and physical domain score showed normal distributions, but cognitive (*p* < 0.01), behavioral (*p* < 0.01), and affective (*p* < 0.01) domain scores did not. As only physical domain data were normally distributed, multivariate analyses were not used to examine group difference. Levene's Tests showed homogeneity of variances for gender, grade, SES, weight status, and race in CAPL-2 composite score and physical domain score.

**Table 4:** PL statistics by gender, grade, SES, BMI, race, and ethnicity.

**Grouping variables**	**Statistics**	**CAPL composite**	**Cognitive domain**	**Physical domain**	**Behavioral domain**	**Affective domain**
		**0 **~** 100**	**0 **~** 10**	**0 **~** 30**	**0 **~** 30**	**0 **~** 30**
Male	M (SD)	62.48 (12.25)	6.50 (1.91)	20.73 (4.66)	10.74 (6.63)	24.68 (4.90)
	*N*	67	89	96	69	89
Female	M (SD)	59.51 (12.05)	7.23 (1.68)	17.59 (5.33)	10.02 (5.71)	23.57 (4.59)
	*N*	90	105	111	95	104
	ΔM	2.97	−0.73	3.14	0.73	1.11
	d	0.20	−0.32^**^	0.52^***^	0.09	0.19^*^
Sixth grade	M (SD)	61.74 (11.35)	6.54 (1.81)	19.82 (5.09)	9.97 (5.86)	24.42 (4.65)
	*N*	80	110	118	86	106
Seventh grade	M (SD)	59.78 (13.00)	7.36 (1.75)	18.03 (5.32)	10.71 (6.39)	23.66 (4.88)
	*N*	77	84	89	78	87
	ΔM	1.96	−0.81	1.80	−0.74	0.76
	d	0.13	−0.37^**^	0.28^*^	−0.10	0.13
Free and reduced-price meal	M (SD)	57.70 (11.14)	6.75 (1.72)	18.66 (5.20)	8.74 (5.57)	23.37 (4.89)
	*N*	77	101	106	81	101
Self-paid	M (SD)	63.74 (12.48)	7.06 (1.92)	19.46 (5.31)	11.87 (6.24)	24.86 (4.51)
	*N*	80	93	101	83	92
	ΔM	−6.04	−0.31	−0.81	−3.14	−1.49
	d	−0.43^**^	−0.14	−0.13	−0.44^**^	−0.26^*^
Underweight	M (SD)	66.25 (10.94)	6.50 (1.05)	23.81 (2.36)	10.34 (9.71)	23.83 (4.37)
	*N*	5	6	6	5	6
Normal weight	M (SD)	64.08 (11.67)	7.14 (1.85)	20.54 (4.76)	11.35 (6.34)	24.99 (4.48)
	*N*	91	112	121	94	111
O & O	M (SD)	55.48 (11.47)	6.50 (1.78)	16.53 (5.00)	9.05 (5.31)	22.95 (4.96)
	*N*	58	70	76	60	70
	ΔM (NW–O&O)	8.60	0.64	4.01	2.30	2.03
	d	0.61^***^	0.29^*^	0.68^***^	0.31^*^	0.36^**^
All other races	M (SD)	58.79 (12.46)	6.42 (2.06)	18.74 (5.61)	10.40 (6.13)	23.10 (4.50)
	*N*	31	39	57	32	38
Black/African American	M (SD)	59.68 (11.97)	6.59 (1.64)	19.03 (5.21)	8.35 (6.10)	24.11 (4.87)
	*N*	49	67	64	53	66
White	M (SD)	62.28 (12.18)	7.34 (1.76)	19.28 (5.09)	11.62 (5.82)	24.48 (4.77)
	*N*	77	88	86	79	89
	ΔM (B–W)	−2.60	−0.75	−0.25	−3.26	−0.37
	d	−0.18	−0.36^**^	−0.04	−0.44^***^	−0.06
Hispanic/Latino	M (SD)	57.62 (12.54)	6.30 (2.14)	19.36 (6.14)	10.09 (5.50)	22.88 (5.67)
	*N*	10	12	11	11	12
Non-hispanic/Latino	M (SD)	60.99 (12.18)	6.94 (1.80)	19.17 (5.19)	10.34 (6.16)	24.16 (4.70)
	*N*	147	182	175	153	181
	ΔM	−3.38	−0.64	0.20	−0.25	−1.28
	d	−0.22	−0.26	0.03	−0.04	−0.19

*CAPL, Canadian Assessment of Physical Literacy; M, mean; N, number; SD, standard deviation; ΔM, mean differences; NW, Normal Weight; B, Black; W, White; O&O, Overweight and Obese;*

*** *p < 0.001;*

** *p < 0.01;*

* *p < 0.05*.

### Differences Across Demographic Variables at Baseline

Univariate and non-parametric analyses revealed statistically significant differences in (a) CAPL-2 composite score by SES and IBM, (b) cognitive and physical domain scores by gender, grade, BMI, and race; (c) behavioral domain score by SES, BMI, and race; and (e) affective domain score by gender, SES, and BMI. Table 4 shows the detailed results for these group differences.

### PL Journey in Light of Receiving the SDT-Based Pedagogical Workshop

Students' PL development trajectories were informed by quantitative and qualitative data based on the subsample. Of the original 49 students, 11 students dropped out of the workshop, which led to a retention rate of 77.6%. There were 41, 39, 42, and 35 students participated in the session #1, #2, #4, and #4, respectively; and zero, three, six, 16, and 24 students completed zero, one, two, three, and four workshop sessions respectively. Table 5 shows the descriptive results from CAPL-2 data for pre and posttests. Significant group [*F*_(1,27)_ = 37.60, *p* < 0.01, $\eta_{p}^{2}$ = 0.58] and time by group interaction [*F*_(1,27)_ = 14.94, *p* < 0.01, $\eta_{p}^{2}$ = 0.36] effects were observed in behavioral domain. In the high-performing PL group, the CAPL-2 composite score, and physical, behavioral, and affective domain scores decreased (*g*: 0.02–0.53) over time, but cognitive domain score increased (*g* = 0.15). In the low-performing PL group, CAPL-2 composite score and four domain scores increased over time (*g* = 0.34–0.69).

**Table 5:** Descriptive results of CAPL assessments for the students intervened by workshop.

**Time**	**Group**	**Statistics**	**CAPL composite**	**Cognitive domain**	**Physical domain**	**Behavioral domain**	**Affective domain**
			**0 **~** 100**	**0 **~** 10**	**0 **~** 30**	**0 **~** 30**	**0 **~** 30**
Pretest	High PL (*n* = 14)	M (SD)	79.04 (4.94)	7.93 (1.33)	24.42 (3.49)	18.67 (5.26)	27.98 (2.07)
	Low PL (*n* = 24)		51.90 (10.88)	6.12 (1.65)	17.62 (5.24)	7.29 (3.97)	21.03 (5.59)
Posttest	High PL (*n* = 11–14)	M (SD)	75.78 (8.91)	8.15 (1.72)	23.73 (3.76)	15.88 (5.24)	27.94 (2.49)
	Low PL (*n* = 20–22)		59.32 (10.47)	6.95 (1.96)	19.33 (4.12)	8.90 (5.26)	22.90 (5.23)
Post-pretest (high PL group)		*ΔM*	−3.26	0.23	−0.68	−2.79	−0.04
		Hedges'*g*	−0.46	0.15	−0.19	−0.53	−0.02
Post-pretest (low PL group)		*ΔM*	7.42	0.83	1.71	1.61	1.86
		Hedges'*g*	0.69	0.46	0.36	0.35	0.34

*CAPL, Canadian Assessment of Physical Literacy; M, mean; N, number; SD, standard deviation; ΔM, mean differences*.

Forty-four and 43 students attended the focus group interviews at pretest and posttest, respectively, while 33 participated in both (retention rate = 67.4%). Three themes emerged from the data analysis: (1) PA pattern (type, frequency, and intensity), (2) motivation, (3) and barriers.

#### PA Type

Of the 33 interviewees, 19 demonstrated more diverse PA choices at post-interview than pre-interview, while five interviewees showed less diverse choices and nine showed no change. The high- and low-performing PL groups reported approximately the same amount of PA types at both interviews, but both groups reported more types of physical activities at the post-interview than the pre-interview. The most commonly reported PA types were gymnastics, track, swimming, basketball, volleyball, baseball, walking, and chasing a dog. Few students reported engaging in planned exercises.

#### PA Frequency and Intensity

When interviewees were prompted to respond the number of days they performed physical activities in the past 2 weeks, 11 reported more frequent PA participation at post-interview than at the pre-interview, five reported less frequent participation, and the rest reported no change. The participation frequency ranged from once per week to seven days per week. Most of the high-performing students (60.0% at pre- vs. 76.9% post-interviews) maintained a high weekly participation frequency (≥5 days per week); while increased percentage of low-performing students increased participation frequency (from 53.8 to 66.7%). For PA intensity, the number of interviewees from low-performing PL group reported decrease in light intensity PA participation (42.9% vs. 6.3%), and increase in vigorous intensity PA participation (28.6 vs. 50.0%). The high-performing PL group reported decrease in vigorous PA participation at post-interview (66.7 vs. 77.8%).

#### Motivation

More interviewees reported higher intrinsic (count: 25 vs. 21), extrinsic (count: 22 vs. 18), and combined motivation (count: 18 vs. 9) for PA at post-interview than pre-interview. The frequently mentioned extrinsic motives included (a) for health benefits and (b) for being social with peers, which were focused content of the pedagogical workshop. After receiving the workshop, several interviewees internalized these two reasons of being active. For example, a girl in the high-performing PL group at pre interview stated that “it made me feel good about myself… just for fun.” She was more articulate at the post-interview, where she stated “it's a challenge… I love the routine, I love the people on the cheer team. … I do this with my friends… I like it because it's just fun to do it… it's good for my health, and it's fun in general.” A boy from the low-performing PL group reported his motivation for being active is because he likes “playing baseball,” enjoys “practice a lot,” and likes “these activities that are competitive form.” At the post interview, he explained that he likes “playing outside with [his] friends and doing active stuff so when [he] get[s] older [he] can be in shape.”

#### Barriers

We tallied the counts of perceived barriers to PA participation across the four PL domains at both interviews. The participants reported 67 and 99 barriers to PA participation across the four PL domains at pre- and post-interviews, respectively. The participants who reported “no barriers” increased from 23 to 28. Most barriers did not cast real blockade to PA participation. At pre-interview, most of the barriers were reported in the physical (count = 28) and behavioral (count = 17) domains, followed by affective (count = 12) and cognitive (count = 10) domains. At post-interview, the perceived barriers were mostly in the physical domain (count = 44), affective (count = 31), and behavioral (count = 20) domains; while cognitive domain (count = 4) saw the least number of barriers. The frequently mentioned physical barriers included injuries, body overheat, time/schedule conflict, and lack of skill; while the cognitive domain barriers were mostly reflected by lack of knowledge. These barriers were recognized similarly in the low- and high-performing groups. Frequently perceived affective domain barriers were social appraisal and lack of confidence, especially among the low-performing group students. Social appraisals such as feeling embarrassed, criticized, or judged in a group situation were a concern for two boys and two girls in the low-performing PL group. Here are several interview quotes: “I feel like I'm gonna miss the ball, I'm gonna be laughed,” “I sometimes I can't take the humiliation,” “I was really upset because I wasn't sure what to do, because I didn't want people judging me or me judging myself if didn't make it…I just didn't want to feel that disappointment.”

## Discussion

This study addressed aimed: (a) to characterize middle school students' PL development and distribution patterns by sociodemographic factors, and (b) to capture students' PL journeys in light of receiving a SDT-guided pedagogical workshop, with a long-term vision on PYD. The findings bear significant implications to the research and practice related to PL and PYD in and beyond school. The findings are discussed below.

A major finding of this study is that the middle school students' holistic PL level [Healthy Active Living and Obesity Research Group (HALO), 2017; Tremblay et al., 2018a] was found to be low or at the “progressing stage” [Healthy Active Living and Obesity Research Group (HALO), 2017] for both genders, warranting the need for purposeful intervention. This observation is in line with findings from other studies across North America, in Canada in particular (Bélanger et al., 2018; Dutil et al., 2018; Tremblay et al., 2018b). We observed significant group differences in PL and/or PL domains by gender, SES, BMI, race & ethnicity, and grade. Specifically, boys scored higher in overall PL, physical and affective domains, but lower in cognitive domain than girls. The higher scores in boys for overall PL (Longmuir et al., 2015; Bélanger et al., 2018; Tremblay et al., 2018b) and physical domain (Bélanger et al., 2018) are consistent with previous observations. The physical domain of the CAPL-2 is represented by movement skill and health-related fitness, two areas that boys often outperform girls. PE curricula and instruction should continue embracing developmentally appropriate content and strategies to foster girls' movement skills and health-related fitness. In addition, boys also scored higher in the affective domain but lower in the cognitive domain than girls, which are also in line with prior research (Chen et al., 2017; Bélanger et al., 2018). Lack of motivation in adolescent girls is a significant barrier to PA participation (Motl et al., 2003; Allender et al., 2006). Compared to girls, boys scored lower at the knowledge test, which demands pedagogical attention as well (Chen et al., 2017; Liu and Chen, 2020b).

Grade differences were observed in cognitive and physical domain scores, with sixth graders scoring higher in the physical domain but lower in the cognitive domain than seventh graders. Higher knowledge about PA and fitness in seventh grade than sixth grade appears to be consistent with previous observations (Law et al., 2018; Tremblay et al., 2018b; Chen et al., 2019; Zhang et al., 2019); however, prior research often reported lower scores for the physical domain (fitness and skills) constructs in older adolescents (Tremblay et al., 2018b). Such mixed reports of age or grade differences need further empirical research investigation.

Additionally, we found that the higher SES group showed significantly higher scores in overall PL, and behavioral and affective domains than the lower SES group. They also showed higher mean scores in the cognitive and physical domains, though not statistically significant. Although this was the first study that has investigated SES-based difference in PL development, prior research has examined SES-based difference in relevant constructs. For example, prior studies found higher SES to associate with more favorable PA behavior (Kantomaa et al., 2007; Drenowatz et al., 2010). These findings reinforce that learning and health outcomes of lower SES groups remain a concern that demands purposeful intervention. To promote lower SES children's PA, motivation, physical competence, and learning, it is important to provide them with safe (King and Ling, 2015) and need-supportive environments (Shannon et al., 2018), where we facilitate the opportunities of PA such as game play and outdoor activity (Johnstone et al., 2019).

Compared with the overweight/obese group, the group with healthy BMI scored higher in overall PL (*d* = 0.61) and all four PL domains (*d*: 0.29–0.68). This finding is consistent with Delisle Nyström et al.'s (2018) observations. As evidenced by epidemiology research, having an abnormal BMI may be detrimental to health (Bischoff et al., 2017). Overweight or obesity is a barrier to PA participation (Bischoff et al., 2017). These findings indicate the need for offering tailored instructions for students with unhealthy BMI to improve their PL development.

By race, the cognitive and behavioral domains of the CAPL-2 assessments favored White over Black students. The cognitive domain assessment included knowledge and understanding about PA, fitness, and health. The finding is similar to findings from two previous studies that used the PE Metrics written test (Chen et al., 2017; Zhang et al., 2019). Possessing sufficient knowledge and understanding about PA and fitness is not only an essential learning goal of quality PE [Society of Health Physical Educators (SHAPE) America, 2013], but also a needed competency area of active living (Chen et al., 2017; Liu and Chen, 2020b). The lower score in cognitive and behavioral domains in Black students highlight the need for more attention to race-related pedagogy in PE. This also holds true for culturally relevant and ethnicity-related pedagogy; as PL composite score and scores in the cognitive, physical, and affective domains all favored the non-Hispanic group. These results indicate the need to purposely promote racial (e.g., Blacks) and ethnic minority (e.g., Hispanics) students' PL development in and outside of PE.

### PL Journey in Light of Receiving the Workshop

The other significant finding of this study is that the students' PL development trajectories varied interpersonally. Our quantitative results demonstrated significant increases in PL and some PL domains, as students received the SDT-based pedagogical workshop. The intervention was delivered every 2 weeks across 8 weeks. Specifically, the net gain of CAPL-2 composite score from pre-test to post-test was 3.59 (5.8%). The positive change in overall PL was mainly contributed by improvements shown in the cognitive (*d* = 0.24), physical (*d* = 0.25), and affective (*d* = 0.26) domains. Consistent with previous studies, knowledge and understanding (Demetriou et al., 2015; Kiez, 2015; Chen et al., 2019) and affective domain variables (Sánchez-Oliva et al., 2017; Wainwright et al., 2018) can be improved through school-based interventions. The PL changes, in small effect size, may be due to the possibility that PL development is a life journey and the eight-week workshop could only render small effects. Notably, the low-performing PL group students demonstrated improvements in overall PL and all four domains (Hedges'*g*: 0.34–0.69). In contrast, the high-performing PL group students' scores in overall PL and three PL domains (i.e., physical, behavioral, and affective) showed decline at the posttest compared to pretest with small to medium effect sizes; although they also showed improvement in the cognitive domain (small effect size). The little to no changes of PL scores in the high-performing PL group may be attributable to the fact that the CAPL-2 has maximum scores on PL and PL domains which limited its validity to accurately capture the actual PL level of these high-performing students. However, the significant improvement in the low-performing PL group suggests that learning did occur. Furthermore, the gap for overall PL score between high- and low-performing PL group was narrower at posttest (Δ*M* = 16.64) than pretest (Δ*M* = 27.14). Low-performing PL students' greater gain of the PL score indicates that they developed their PL from the “beginning” stage to the “developing” stage [Healthy Active Living and Obesity Research Group (HALO), 2017]. The changes of PL level in both low and high-performing groups indicate the gained competencies in cognitive, affective, physical, and behavioral domains of PL, in light of receiving the SDT-based workshop.

Interview data further substantiate the positive developing trajectories of PL as shown in the noticeable changes in PA patterns, enjoyment, and barriers. Overall, the workshop attendees showed more diverse and conducive PA patterns. They voiced more barriers to PA participation in the post-interview than the pre-interview. However, we interpret this result as a positive change, where the participants have improved their awareness of physical, cognitive, affective, and behavioral barriers. These perceived barriers did not restrain them from engaging in physical activities of various types, frequency, and intensity. The positive gains shown in the focus group interview data (also evidenced by the CAPL-2 gain scores) could be attributable to the SDT-based pedagogical workshop (Vasconcellos et al., 2020). Each workshop session consisted of two modules: motivational and informational modules, which provided needs support for students' to perceive competence, autonomy, and relatedness (Deci and Ryan, 1985). The motivational module emphasized and facilitated affective development to help students recall and share their fun experiences over physical activities in the past 2 weeks, which might have nurtured the motivation (affective domain of PL) to be more intrinsically or internally oriented. The sharing part enhanced social processing and belonging with peers (relatedness). The informational module focused on enhancing students' competence and perceived competence in cognitive and partially physical domains by teaching PA, fitness, and motor skill knowledge and coping strategies. Language of workshop materials were rephrased to become readable to middle school students. As a result, the significant increases in cognitive and affective domains were observed in the quantitative data, which might have been in response to the pedagogical workshop offerings (e.g., tailored information, activities, and experiences). However, changes in the physical and behavioral domains of CAPL-2 assessments were not significant. This might partially be due to the lack of direct trainings for physical fitness, skills, and behavior change within these short workshops. Unlike the quantitative data, the focus group interview data demonstrated a favorable change of PA patterns, especially among the students in the low-performing PL group. The interview data also showed that the students who received the workshop had positive experiences which improved their learning, attitude, and behaviors. However, it should be acknowledged that the workshop was only a short addition to the PE program. The participants' PL development and physical embodiment may be influenced by factors both in PE and out-of-school contexts ranging from organized sport and PA programs, playing with friends after school, or non-profit programs such as Girl Scouts and Girls on the Run. As articulated by Allan et al. (2017), there are ample opportunities from out-of-school settings to foster PL and PYD. These programs tend to have optimal potential to achievement such development when following the 5C PYD model (i.e., competence, confidence, connection, and character; Lerner, 2009). These additional experiences beyond the PE context may interact with the PE experiences (including the pedagogical workshop) enhancing students' PL development.

This is to our knowledge the first empirical study that has examined middle school students' PL development within the context of PE in the U.S. The study has several strengths including the sequential mixed methods interventional design, use of valid measures, and robust data analyses. However, the study also has several limitations. First, due to time and human resource limitation, we were unable to gather posttest data from a control group. Although the mixed methods data showed gains in PL, especially in the low-performing PL group, the observed gains may attribute to the pedagogical workshop intervention, time/maturation, familiarity of the test or inadvertent teaching to the test. However, the interview questions were piloted prior to data collection and interviews data were triangulated with data collected from other reliable sources (e.g., CAPL-2, observations). It also should be acknowledged that this study took time from regular PE (though time and interruption was deemed insignificant) which might have intuitively weakened the argument that the gains were from the pedagogical workshop intervention. Second, despite the relatively large sample size, the sample originated from students enrolled in one single public middle school located in the Southeastern U.S. Thus, findings of this study can only be generalized to students and schools of similar characteristics. Third, students' SES was determined by their eligibility for free and reduced-price meal, which only informs the economic aspect of SES, while other aspects including educational and occupational factors were not assessed.

## Conclusions

This study characterized students' PL and PL domains through a pilot, SDT-based pedagogical workshop intervention in middle school PE. PE is an essential setting to influence the growth and development of school-aged children and adolescents. Effective and purposeful PE curriculum and instruction can contribute to PL and PYD (Wright and Li, 2009; Weiss, 2011; Holt et al., 2012). The students' PL was found to be at the progressing stage, which varied by sociodemographic factors, showing their vulnerability in achieving lifelong PA participation. The differences identified in this study by gender, SES, BMI, race and ethnicity, and grade warrant future specifically targeted (rather than generic) intervention. The SDT-based pedagogical workshop was associated with favorable changes in PL development. Future studies may use SDT to guide students' navigation for PL development in PE. Future work should also examine the effect (and implementation) of opportunities both in (e.g., PE) and outside of schools (e.g., youth sports programs) to ultimately advance PYD.

## Data Availability Statement

The raw data supporting the conclusions of this article will be made available by the authors, without undue reservation.

## Ethics Statement

The studies involving human participants were reviewed and approved by Institutional Review Board, Office of Research and Economic Development, Louisiana State University. Written informed consent to participate in this study was provided by the participants' legal guardian/next of kin.

## Author Contributions

SC and YL jointly designed the study. YL collected, processed, analyzed the data, and drafted the manuscript. SC contributed to data processing and analysis. YL and SC revised the manuscript. All authors contributed to the article and approved the submitted version.

## Funding

This work was supported by 2019 College of Human Sciences and Education (CHSE) Dean's Circle Grant Program of Louisiana State University. The design and execution of this study was supported by the 2019 College of Human Sciences and Education Dean's Circle Grant Program of Louisiana State University. The publication of this article was supported by the Eunice Kennedy Shriver National Institute of Child Health and Human Development of the National Institutes of Health under award number R21HD090513.

## Conflict of Interest

The authors declare that the research was conducted in the absence of any commercial or financial relationships that could be construed as a potential conflict of interest.

## Publisher's Note

All claims expressed in this article are solely those of the authors and do not necessarily represent those of their affiliated organizations, or those of the publisher, the editors and the reviewers. Any product that may be evaluated in this article, or claim that may be made by its manufacturer, is not guaranteed or endorsed by the publisher.
